# Supplementary material for: Disentangling pontine fiber geometry and microstructure in ARSACS using advanced MRI
Source: Neuroimage Clin. 2026 Sep 21;52:104066. doi: 10.1016/j.nicl.2026.104066 (PMC13636084; doi:10.1016/j.nicl.2026.104066)
Supplement: Supplementary file 1 [file mmc1.docx]

# Supplementary

*Figure S 1: Microstructural profile analysis: Average patient and control profiles along segments of the cortical spinal tract (right) (CST_R_). The shaded regions correspond to the standard deviation between subjects, the shaded dots correspond to segments that overlap at greater than 40% between the two tracts, and red dots correspond to segments where patient and control profiles significantly differ (p<0.01).*

*Figure S 2: Whole-tract boxplots and profile analysis of T2_e_ and D_e⊥_ in the left (CST_L_) and right (CST_R_) cortical spinal tract, the middle cerebellar peduncles (MCP) and transverse pontine fibers (TPF). Boxplots: (**) significant at p<0.01, (*) significant at p<0.05, (ns) not significant and red dots correspond to segments where patient and control profiles significantly differ (p<0.01).*

*Figure S 3: Spearman’s correlation between MT_sat_ and De_perp_ and T2_e_ across patients and controls. Significant correlations (p<0.05) are shown in red.*

*Figure S 4: Cohen’s d effect sizes and 95% confidence intervals (CI) for microstructural parameters in the CST and MCP+TPF. The limit for small (d<0.2; dotted blue line) and large (d>0.8; dotted black line) effects are shown. The red dots correspond to FDR-corrected significant segments (p<0.01) from the profile analysis.*

*Figure S 5: Quality of fit: Correlation of rotational invariants of predicted and measured signals for a presentative subject. On average, the correlation coefficient (r) was 0.96 +/- 0.03 and the bias was 2.8+/-3.5%.*

(a) Anatomical volumes

| **Metric** | **Group Effect (p)** | **Age Effect (p)** | **Sex Effect (p)** | **Significant interactions** |
| --- | --- | --- | --- | --- |
| **ICV** | **0.012** | 0.125 | 0.192 | Group×Age×Sex (**0.036**) |
| **White Matter** | **<0.001** | 0.908 | 0.052 | None |
| **WM/ICV** | **<0.001** | **0.047** | 0.204 | Age effect |
| **Cerebellum** | **<0.001** | 0.945 | **0.042** | Sex effect |
| **Cerebellum/ICV** | **<0.001** | 0.123 | 0.162 | None |
| **Pons** | **0.005** | 0.276 | 0.178 | None |
| Pons/ICV | 0.14 | 0.103 | 0.654 | None |

(b) Tract volumes

| **Metric** | **Group Effect (p)** | **Age Effect (p)** | **Sex Effect (p)** | **Significant interactions** |
| --- | --- | --- | --- | --- |
| **CSTL/ICV** | **<0.001** | 0.99 | 0.327 | None |
| **CSTR/ICV** | **<0.001** | 0.286 | 0.24 | None |
| MCP/ICV | 0.542 | 0.264 | 0.693 | None |
| **TPF/ICV** | **0.0015** | 0.117 | 0.287 | Group×Age **(0.029)** |

(c) Microstructural parameters

| **Metric** | **Group Effect (p)** | **Age Effect (p)** | **Sex Effect (p)** | **Significant Interaction(s)** |
| --- | --- | --- | --- | --- |
| **CSTL T2a** | **<0.001** | 0.344 | 0.821 | None |
| **CSTR T2a** | **0.012** | 0.758 | 0.79 | None |
| TPF T2a | 0.152 | 0.847 | 0.381 | Group×Age (**0.042**) |
| MCP T2a | 0.558 | 0.842 | 0.885 | None |
| **CSTL f** | **<0.001** | 0.136 | **0.01** | Age×Sex (**0.022**) |
| **CSTR f** | **<0.001** | 0.753 | 0.122 | Age×Sex (**0.008**) |
| TPF f | 0.289 | 0.42 | 0.393 | None |
| **MCP f** | **<0.001** | 0.526 | 0.787 | Group×Age (**0.046**) |
| **CSTL FA** | **0.007** | 0.794 | 0.601 | None |
| **CSTR FA** | **0.02** | 0.807 | 0.858 | None |
| **TPF FA** | **0.041** | 0.857 | 0.267 | None |
| MCP FA | 0.113 | 0.694 | **0.02** | None |
| **CSTL MTsat** | **0.002** | 0.709 | 0.48 | Group×Age (**0.006**) |
| **CSTR MTsat** | **0.01** | 0.749 | 0.558 | Group×Age (**0.033**) |
| **TPF MTsat** | **0.033** | 0.948 | 0.198 | None |
| MCP MTsat | 0.934 | 0.616 | 0.865 | None |

*Table S 1: Sensitivity analysis using ANCOVA models that included age and sex as covariates for (a) volumetric data (b) tract volumes and (c) microstructural parameters. Significant interactions (p<0.05) are in bold*

(a) SARA with Anatomical volumes

| **Metric** | **SARA p** | **Age p** | **Sex p** | **Interpretation** |
| --- | --- | --- | --- | --- |
| ICV | 0.166 | 0.879 | 0.474 | No association |
| White Matter | 0.166 | 0.876 | 0.473 | No association |
| WM/ICV | 0.169 | 0.883 | 0.484 | No association |
| Cerebellum | 0.165 | 0.876 | 0.47 | No association |
| Cerebellum/ICV | 0.169 | 0.884 | 0.483 | No association |
| Pons | 0.166 | 0.879 | 0.474 | No association |
| Pons/ICV | 0.169 | 0.884 | 0.483 | No association |

(b) SARA with Tract Volumes

| **Metric** | **SARA p** | **Age p** | **Sex p** | **Interpretation** |
| --- | --- | --- | --- | --- |
| CSTL/ICV | 0.989 | 0.199 | 0.503 | No association |
| CSTR/ICV | 0.097 | 0.845 | 0.949 | No association |
| MCP/ICV | 0.986 | 0.548 | 0.393 | No association |
| TPF/ICV | 0.243 | 0.097 | 0.938 | No association |

(c) SARA with Microstructural parameters

| **Metric** | **Group Effect (p)** | **Age Effect (p)** | **Sex Effect (p)** | **Interpretation** |
| --- | --- | --- | --- | --- |
| CSTL T2a | 0.935 | 0.637 | 0.871 | No association |
| CSTR T2a | 0.576 | 0.903 | 0.81 | No association |
| TPF T2a | 0.75 | **0.043** | **0.033** | Age and sex effects only |
| MCP T2a | 0.805 | 0.941 | 0.861 | No association |
| CSTL f | 0.769 | 0.641 | 0.213 | No association |
| CSTR f | 0.261 | 0.61 | 0.622 | No association |
| TPF f | 0.996 | 0.401 | 0.147 | No association |
| MCP f | 0.761 | 0.221 | 0.952 | No association |
| CSTL FA | 0.667 | 0.951 | 0.632 | No association |
| CSTR FA | 0.949 | 0.883 | 0.773 | No association |
| TPF FA | 0.525 | 0.374 | 0.179 | No association |
| MCP FA | 0.961 | 0.903 | 0.619 | No association |
| **CSTL MTsat** | **0.047** | **0.002** | **0.023** | Significant SARA association |
| CSTR MTsat | 0.123 | **0.029** | 0.121 | Age effect only |
| TPF MTsat | 0.355 | 0.282 | 0.086 | No association |
| MCP MTsat | 0.723 | 0.849 | 0.76 | No association |

*Table S 2: Associations with clinical severity (SARA) using a general model that included age and sex for (a) volumetric data (b) tract volumes and (c) microstructural parameters. Significant interactions (p<0.05) are in bold*
